# Treatment outcomes of postoperative mediastinitis in cardiac surgery; negative pressure wound therapy versus conventional treatment

**DOI:** 10.1186/1749-8090-7-67

**Published:** 2012-07-11

**Authors:** Hayati Deniz, Gokhan Gokaslan, Yavuz Arslanoglu, Ozerdem Ozcaliskan, Gokalp Guzel, Alptekin Yasim, Hasim Ustunsoy

**Affiliations:** 1 Gaziantep University Medical Faculty, Department of Cardiovascular Surgery, 27310, Sehitkamil, Gaziantep, Turkey

**Keywords:** Cardiac surgery, Sternal wound infection, Negative pressure wound therapy

## Abstract

**Background:**

The aim of the present study is to compare negative pressure wound therapy versus conventional treatment outcomes at postoperative mediastinitis after cardiac surgery.

**Methods:**

Between January 2000 and December 2011, after 9972 sternotomies, postoperative mediastinitis was diagnosed in 90 patients. The treatment modalities divided the patients into two groups: group 1 patients (n = 47) were initially treated with the negative pressure wound therapy and group 2 patients (n = 43) were underwent conventional treatment protocols. The outcomes were investigated with Kaplan-Meier method, log-rank test, Student’s test and Fisher’s exact test.

**Results:**

The 90-days mortality was found significantly lower in the negative pressure wound group than in the conventionally treated group. Overall survival was significantly better in the negative pressure wound group than in the conventionally treated group.

**Conclusion:**

Negative pressure wound therapy is safe and reliable option in mediastinitis after cardiac surgery, with excellent survival and low failure rate when compared with conventional treatments.

## Background

Infection of the sternotomy wound is a potentially devastating and sometimes lethal complication following cardiac surgery. Although the incidence of post-cardiotomy mediastinitis rate has been variously reported as between 0.8 and 5.0%, the mortality rate varies between 19% and 29% in different series of adult cardiac surgical patients [1-5]. Established treatment may involve a combination of debridement, packing, delayed closure, plastic reconstruction, re-wiring and irrigation together with antibiotherapy dependent on the severity of infection. However, such treatments can be complex, invasive and prolong the hospitalization particularly when the primary method fails. These conventional wound healing techniques may be a combination of different procedures, but there is still a lack of consensus for the optimal surgical management. Conventional treatment has disadvantages such as destabilization of the sternum, prolonged immobilization and concomitant infections, that may complicate this fatal treatment period [2-4]. Application of negative pressure by controlled suction through a porous dressing has emerged as a simple and effective treatment for a wide spectrum of wounds [6]. Several studies have been reported with promising results with the use of vacuum-assisted closure (VAC) therapy in poststernotomy mediastinitis that are also called deep sternal wound infections (DSWI) [1,7-9].

The aim of the present study is to compare the clinical outcomes and survival in our 90 patients underwent negative pressure wound therapy (NPWT) or conventional treatment for mediastinitis after cardiac surgery.

## Methods

Between January 2000 and December 2011, 9972 sternotomies were performed in patients underwent cardiac surgical procedures at our cardiovascular surgery department. After a retrospective study design the study protocol was approved by the ethics committee for clinical research at our institution (Reference number: 22.11.2011/238). Only patients with mediastinitis those are consisting of sternum and presternal tissue infection were included in the present study. Patients undergoing thoracic aortic surgery extending to descending aorta, heart transplantation, congenital heart surgery and patients presenting signs of infection but with negative substernal tissue cultures or without mediastinitis were excluded from the study.

In 90 patients (0,9%) postoperative mediastinitis was diagnosed, based on the guidelines of the US Centre for Disease Control and prevention [10]. Wound classification was defined according to the suggestions of El Oakley and Wright [11]. The diagnosis required at least one of the following criteria: (1) an organism was isolated from culture of mediastinal tissue or fluid; (2) evidence of mediastinitis was seen during operation; or (3) one of the following conditions, chest pain, sternal instability (detachment), or fever (>38°C) was present and there was either purulent discharge from the mediastinum or an organism isolated from blood culture or drainage culture from the mediastinal area.

Patients’ characteristics were analyzed retrospectively and according to the treatment modality, patients were divided into two groups: group 1 patients (n = 47) were initially treated with the NPWT and group 2 patients (n = 43) were underwent conventional treatment protocols. Choice of these protocols differed by time. Almost all patients with mediastinitis were treated by conventional protocols until 2003. By development of NPWT techniques; we increasingly used this protocol after 2003. By the time of initial diagnosis or suspicion of mediastinitis, bacteriological cultures of wound secretions were routinely taken from all patients and subsequently both protocols were continued until the consecutive bacteriologic cultures became negative. Afterwards surgery for primary closure of the sternum was performed.

### Conventional treatment

Between January 2000 and December 2003, 43 consecutive patients were diagnosed as having poststernotomy mediastinitis. The technique of sternal excision and closure has been described previously [12]. The patients in this group underwent initial surgical revision with debridement of the presternal infected tissue and sternal edges where appropriate. This revision included removal of fibrins, necrotic tissue, and sternal wires. The surgical procedures which performed (rewiring, open dressings, closed irrigation or pectoral flaps) depended on the clinical condition of the patient and the surgeon’s preference. Pectoral flaps were performed in cooperation with plastic surgeon.

Open dressings consisted of moist saline gauzes in the mediastinum for several days and were changed several times daily in combination with surgical revision. The procedure was concluded with a sterile drape covering the wound. When the wound became clean and there was a bed of fresh granulation tissue, the sternum was rewired.

Closed irrigation was initiated with one or two drains in the mediastinum. The sternum was closed in a standard manner with interrupted steel wires. The wound was also irrigated with normal saline solution, until the infection was considered under control. The drainage tubes were removed in the ward several hours after irrigation had ceased.

### Vacuum-assisted treatment

Between May 2003 and December 2011, 47 consecutive patients with poststernotomy mediastinitis underwent NPWT. The patients in this group underwent initial surgical revision with removal of all sternal wires. The treatment modality was performed using the KCI system (KCI, San Antonio, Texas), with a vacuum pressure between 75 mm and 125 mmHG according to the method described before [13].

This wound-healing technique is based on application local negative pressure to a wound. this is achieved by placing polyurethane foam with an open pore structure of 400–600 μm in the wound. One end of a non-collapsible tube is then connected to the foam and the other end is connected to a vacuum-source in a closed system via connected to a fluid container. The foam and the entire wound are covered with an adhesive drape thus ensuring an air-tight system. Finally, a predetermined, intermittent, negative pressure is applied to the wound. Debridement and exchange of the sponge was performed every 48 to 72 h in the operation room. Substernal tissue cultures were collected for microbiological investigation and determination of the antibiotic resistance pattern. The wound was revised during VAC exchanges with sharp spoon and necrotic bone was removed if necessary, but extensive sternectomy was avoided.

All patients in both groups, prior to their primary operation received standardized preoperative antibiotic prophylaxis with two doses of intravenous cefuroxime 1.5 g, on the day of operation and the first postoperative day. When the DSWI was diagnosed, the antibiotic therapy usually commenced with vancomysin intravenously and continued until the results of the wound cultures became available. The entire panorama of pathogens is presented in Table 1. Thereafter, the antibiotic therapy was adjusted according to bacterial sensitivity and strain. The antibiotic regimen was similar in both groups.

**Table 1:** Culture-verified deep sternal wound infection pathogens

**Bacterial strains**	**VAC therapy**		**Conventional treatment**	
	**n**	**%**	**n**	**%**
*S.aureus*	14	29.8	15	34.9
*E.coli*	4	8.5	6	14
*K.pneumoniae*	1	2.1	-	0
*S. epidermiditis*	9	19.1	8	18.6
Metisin resistance *S. aureus*	12	25.5	10	23.3
*Streptococcus*	-	0	1	2.3
*E.coli + P.aeruginosa*	2	4.3	2	4.7
*P.aeruginosa*	3	6.4	-	0
*A.baumannii*	2	4.3	1	2.3

The sum of percentages exceeds 100% because of rounding-off errors.

*VAC* = vacuum-assisted closure.

*S. aureus = Stafhylococcus aures; E. coli = Escherichia coli; K. pneumoniae = Klebsiella pneumoniae; S. epidermiditis = Stafhylococcus epidermiditis; E.coli = Escherichia coli; P.auroginosa = Pseudomonas aeruginosa; A.baumannii = Acinetobacter baumannii*.

The preoperative variables, including the EuroSCORE [14], were collected from the department’s database (Table 2). EuroSCORE was used to assess the grade of surgical complexity and preoperative status. In addition, risk factors considered relevant to poor wound healing and enhanced risk for sternal detachment, such as diabetes mellitus, obesity, low left ventricular ejection fraction (LVEF), chronic obstructive pulmonary disease (COPD), elderly age, renal failure, emergency surgery, re-do surgery, re-exploration, post perfusion syndrome, prolonged mechanical ventilation, prolonged intensive care unit (ICU) stay, prolonged use of inotropic drugs and immunosuppression therapy [3,4,9,14,15], were collected from patients’ medical records.

**Table 2:** Patients characteristics

**Variable**	**VAC therapy**		**Conventional treatment**		***p*value**
	**n**	**%**	**n**	**%**	
Number of patients	47	52.2	43	47.8	
Sex					
Male	12	25.3	21	48.8	0.003**
Female	35	74.7	22	51.2	0.003**
Surgical procedure					
Isolated or combined CABG procedure	32	68.1	29	67.4	0.262
Isolated valvular procedure	15	31.9	14	32.6	0.302
Diabetes mellitus	15	31.9	14	32.6	0.255
Obesity (BMI ≥ 30)	33	70.2	22	51.2	0.043**
LVEF ≤ 0.30	10	21.3	11	25.6	0.116
NYHA Class III–IV	19	40.4	13	30.2	0.078
COPD	9	19.1	8	18.6	0.298
Age > 65 years	26	55.3	13	30.2	0.004**
Renal failure*	2	4.3	1	2.3	0.065
Emergency surgery	7	14.9	5	11.6	0.122
Re-do surgery	4	8.5	3	6.9	0.190
Re-exploration	9	19.1	5	11.6	0.053
Post perfusion syndrome	13	27.7	10	23.2	0.272
Prolonged mechanical ventilation	21	44.7	16	37.2	0.185
ICU stay > 2 days	19	40.4	20	46.5	0.197
Prolonged use of inotropic drugs	20	42.6	17	39.5	0.177
Immunosuppression	1	2.1	1	2.3	0.344
	Mean	SD	Mean	SD	
CPB time (minutes)	119	33.18	123.1	28.28	0.150
X-clamp time (minutes)	98.2	28.08	99.3	14.14	0.313
Age (y)	67.96	10.47	57.3	14.7	0.004**
EuroSCORE	7.5	3.4	5.0	2.6	<0.001**

*Serum creatinin >1.3 mg/dl.

** < 0.05 is the statistically significance value.

*VAC* = Vacuum-assisted closure; *CABG* = Coronary artery bypass graft; *BMI* = body mass index; *LVEF* = left ventricular ejection fraction; *NYHA* = New York Heart Association; *COPD* = chronic obstructive pulmonary disease; *CPB* = cardiopulmonary bypass.

The sum of percentages exceeds 100% because of rounding-off errors.

### Statistical analysis

During the assessment of the study data, we investigated the distribution of categorical measurements according to the frequency and percentages, while we described our numerical parameters with mean and standard deviations. The survival functions for the conventional treatment group and the NPWT group were calculated using the Kaplan-Meier method. The nonparametric survival functions were then compared using the log-rank test. The two-sample Student’s test was used to evaluate continuous variables. For categorical variables, Fisher’s exact test was applied. The results were evaluated at a significance level of p<0.05. NCSS (Number Cruncher Statistical System), 2007&PASS (Power Analysis and Sample Size), 2008 Statistical Software (Utah, USA) were used for statistical analysis.

## Results

Mean age of patients was 62.86 ± 11.6 and 57 (63.3%) of them were female and 33 (36.7%) of them were male. Primary surgery among the 90 patients presenting with DSWI; 54 (60%) had undergone coronary artery bypass revascularization, 29 (32.2%) isolated valvular procedure and 7 (7.8%) combined valvular and coronary bypass procedures. Demographic data and clinical characteristics of the patients are presented in Table 2. These patients were counted from 9972 sternotomies whom underwent a cardiovascular surgery. The incidence of mediastinitis was found 0.9%, for our 11 years cardiac surgery experience.

The total 90-days mortality was 15,6% (14 patients). The 90-days mortality was found significantly lower in the NPWT group than in the conventionally treated group (8.5%, 4 patient versus 23.2%, 10 patients; *p*<0.05). The cause of all deaths in each group was multiorgan failure caused by severe sepsis. Treatment failure was observed in NPWT and conventional treatment groups 2.1% and 4.7% respectively. These patients were classified as El Oakley class type IV A and type IV B (Table 3) and unfortunately lost in first 45 postoperative day.

**Table 3:** Poststernotomy mediastinitis classification

**El Oakley Class**	**VAC therapy**		**Conventional treatment**	
	**n**	**%**	**n**	**%**
I	5	10.6	4	9.3
II	2	4.3	3	7
IIIA	17	36.1	13	30.2
IIIB	22	46.8	21	49
IVA	1	2.1	1	2.3
IVB	-	0	1	2.3
V	-	0	-	0

The sum of percentages exceeds 100% because of rounding-off errors.

*VAC* = vacuum-assisted closure.

All patients in group 1 underwent sternal rewiring without flap surgery, after 3 consecutive bacteriologic cultures from substernal tissue became negative. In the conventional treatment group pectoral flap surgery was performed in 6.9% (3 patients).

Bacteriologic cultures showed the presence of *staphylococi* in the majority of patients. And the leading pathogenic organism was *S.aureus* for each group. The following organism was metisilin resistance *S.aures*. There was no significant difference between groups when compared the rate of polymicrobial infections (Table 3).

The preoperative variables, including the EuroSCORE and the risk factors relevant to poor wound healing were compared (Table 2); Patients from group 1 (67.96 ± 10.47 years) were significantly older than group 2 (57.3 ± 14.7 years, *p*<0.05; Table 2). Elderly patients those over 65 years old were more frequent in group 1 than in group 2, with a statistically significance (*p*<0.05; Table 2). Patients with BMI over 30 were significantly more in group 1 (70.2%) than in group 2 (51.2; *p*<0.05; Table 2). Female frequency was further in group 1 (74.7%) than in group 2 (51.2, *p*<0.05; Table 2) with a statistically significance. There were no significant difference between groups in terms of other risk factors such as diabetes mellitus, low left ventricular ejection fraction (LVEF), chronic obstructive pulmonary disease (COPD), renal failure, emergency surgery, re-do surgery, re-exploration, post perfusion syndrome, prolonged mechanical ventilation, prolonged intensive care unit (ICU) stay, prolonged use of inotropic drugs and immunosuppression therapy (Table 2).

Median hospital stay for VAC group and conventional group were 26 ± 8, 31 ± 9 respectively and whereas the treatment duration for the conventional treatment group was longer than the VAC group 18 ± 9 days, 24 ± 10 days respectively, without statistically significance.

There was no significant difference in recurrent sternal fistulas between group 1 (1 patient, (2.1%)) and group 2 (2 patients, (4.6%)). The patients were readmitted in the second month of discharged period and the fistulas were debrided under general anesthesia. The fistulas were obliterated completely, without sternectomy in combination with antibiotic therapy.

**Figure 1:**
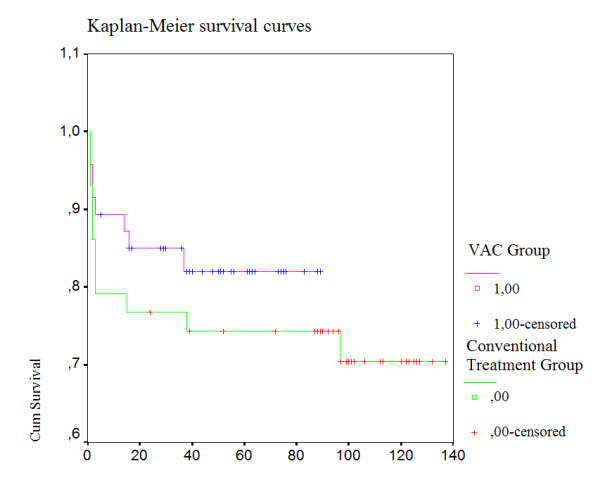
Kaplan-Meier survival curves.

Overall survival was significantly better in the VAC group (p<0.05) than in the conventionally treated group: 91.5% ± 1.2% (n = 43) versus 76.7% ± 1.0% (n = 33) at 1 year, 89.3% ± 2.3% (n = 42) versus 74.4% ± 3.0% (n = 32) at second year and 87.2% ± 2.4% (n = 41) versus 69.8% ± 2.9% (n = 30) at fifth year respectively (Figure1). There were 2 late deaths in the VAC group and 3 late deaths in the conventionally treated group. None of the late deaths were related with ongoing infections in either group.

## Discussion

According to the literature, the incidence of poststernotomy mediastinitis has been variously reported as between 0.8 and 5.0% in different series of adult cardiac surgical patients [1-5,16,17]. Our findings correlate with those of guidelines of the US Centre for Disease Control and prevention [10] and a recent study using the El Oakley classification [11]. In this present study we found the incidence of poststernotomy mediastinitis at a rate of 0,9%, at 9972 sternotomies.

DSWI was initially treated with surgical revision, with or without multiple open dressing changes that have been previously reported with high mortality rates and has major disadvantages: sternal instability which requires mechanical ventilation, prolonged immobilization that increases additional complications as pneumonia, thrombosis and muscular weakening [18]. After some unsatisfying procedures an established method was the use of vascularized soft tissue flaps [19-21]. When Jurkiewicz and colleagues published the first pectoral muscle flap, a lot of studies have been reported varying results with pectoral muscle flaps in poststernotomy mediastinitis [22,23]. Other studies advocate the technique using omentum flaps first described by Lee and coworkers for poststernotomy mediastinitis [24]. These soft tissue flaps has relatively low mortality rate according to some reports, but may be associated with flap-related morbidity [23,25].

VAC therapy is a novel wound healing method. With this method, several advantageous features of conventional treatment are combined. VAC treatment allows open drainage that continuously absorbs the exudate with simultaneous stabilization of the mediastinal cavity and isolation of the wound. This method stimulates granulation tissue formation in combination with increased blood flow in the adjacent tissue [26]. Furthermore, VAC therapy approximates the wound edges and provides a mass filling effect with low degree of surgical trauma [26].

In this study, we retrospectively compared the clinical outcome and survival in 90 patients undergoing VAC therapy or conventional treatment for DSWI after cardiac surgery.

The bacteriologic spectrum, identified in bacteriologic cultures was found similar to other studies with a majority of *S aures* infection (56.7%) and coagulase negative Staphylococcus (18.9%) (Table 1) [27]. Blood cultures were positive in 19 patients (21.1%) and were not predictive for the success rate of the treatment modality. There was also no significant difference in terms of outcome based on organism in our study, which is consistent with the findings from Douville and colleagues [27]. In their study, vacuum-assisted treatment was not available, and patients underwent debridement, drainage and primary sternal reclosure or muscle flap. Overall mortality in their series was reported 12.6%, however 6.3% of them were related with poststernotomy mediastinitis. These results are less than overall mortality of our study (21,1% in our study, of which 15,5% was mediastinitis related) [27].

Catarino and coworkers performed an early, small, retrospectively study and demonstrated a significantly greater number of treatment failures with continuous irrigation compared with VAC therapy [28]. However in our study the number of failures to respond to the VAC treatment was present with a low rate in both of groups. The patients with failures to treatment modalities in both groups were classified as El Oakley class type IV A and type IV B (Table 3). The fistulas were obliterated completely, without sternectomy.

Previous studies have reported that mediastinitis is an independent risk factor with negative influence on long-term survival after coronary bypass graft surgery [3,4,29]. The reason for this negative prognostic effect is not fully understood, but a severe infection can lead other organ malperfusions such as the heart, kidneys and grafts. In our study we observed that VAC group has a significantly better early and long-term survival than the conventionally treated group (Figure1).

Doos and coworker reported a study demonstrating a shorter length of stay and treatment period after VAC therapy. However Sjogren and coworkers did not found similar results with any significant difference in length of stay and treatment duration. But a new study by Simek and coworkers found a particular decrease in the length of hospital stay with statistical significans of P<0.05 when compared with a conventional treatment method (simek). In our study we did not observe any significant difference in length of stay or treatment duration between VAC therapy and conventional treatment [9,30,31].

## Conclusion

Although the population became older, more obese, the increase in proportion of females and the EuroSCORE, during the last decade the incidence of mediastinitis after cardiac surgery did not change. However major changes occurred in number of patients at risk. Although risky patients was found higher in our study which were mostly included in the VAC group, the present study demonstrates that VAC therapy is safe and reliable option in DSWI, with excellent survival and low failure rate when compared with conventional treatments as like as in Assmann A and cowerkers study [32].

## Competing interest

The authors declare that they have no competing interests.

## Authors’ contributions

HD and GG carried out the study design and drafted the manuscript, YA, OO and GG collected patients data, AY performed the statistical analysis, HU participated in the design of the study. All authors read and approved the final manuscript.
